# Repurposing medicinal compounds for blood cancer treatment

**DOI:** 10.1007/s00277-015-2412-1

**Published:** 2015-06-07

**Authors:** Bronagh McCabe, Fabio Liberante, Ken I. Mills

**Affiliations:** Centre for Biomedical Sciences Education, School of Medicine, Dentistry and Biomedical Sciences, Queen’s University Belfast, Belfast, BT9 7AE UK; Blood Cancer Research Group, Centre for Cancer Research and Cell Biology (CCRCB), Queen’s University Belfast, Belfast, BT9 7AE UK

**Keywords:** Blood cancer, Drug repurposing, Review

## Abstract

Drug development is being continuously scrutinised for its lack of productivity. Novel drug development is associated with high costs, high failure rates and lengthy development process. These downfalls combined with a huge demand in blood cancer for new therapeutic treatments have led many to consider the method of drug repurposing. Finding new therapeutic indications for already established drug substances is known as redirecting, repositioning, reprofiling, or repurposing of drugs. Off-patent and on-patent drugs can be screened for additional targets and new indications thus bringing them to clinical trials at a faster pace. This approach offers smaller research groups, such as those that are academic based, into the drug development industry. Drug repurposing can make use of previously published data concerning dosage, toxicology and mechanism of activity.

## Introduction

Cancer is a major cause of death in the world today, and its level of incidence is set to increase globally putting pressure on health services and pharmaceutical companies to develop novel therapies at a competitive pace.

The current model of drug discovering is being continuously scrutinised due to the high failure rate that has been recently associated with it. Finding new therapeutic indications for already established drug substances is known as redirecting, repositioning, reprofiling, or repurposing of drugs [1]. For the purpose of this review, the strategy will be known as repurposing of drugs. This method of developing novel drug treatments is becoming increasingly more attractive as an alternative to the current methods due to the huge reduction in time needed to achieve accreditation and introduce the drug into the market [2].

In the past decades, the number of novel drugs being introduced into the market has had not significantly increased, even with recent advances in technology both informational and biological. The total research and development (R&D) spending for drug discovery worldwide has increased at least 15-fold from 1975; however, the number of new molecular entities (NMEs) approved by the Food and Drug Administration (FDA) in the USA did not show a similar dramatic increase: 26 new drugs approved in 1976 and 41 novel new drugs approved in 2014 [3–5].

**Fig. 1:**
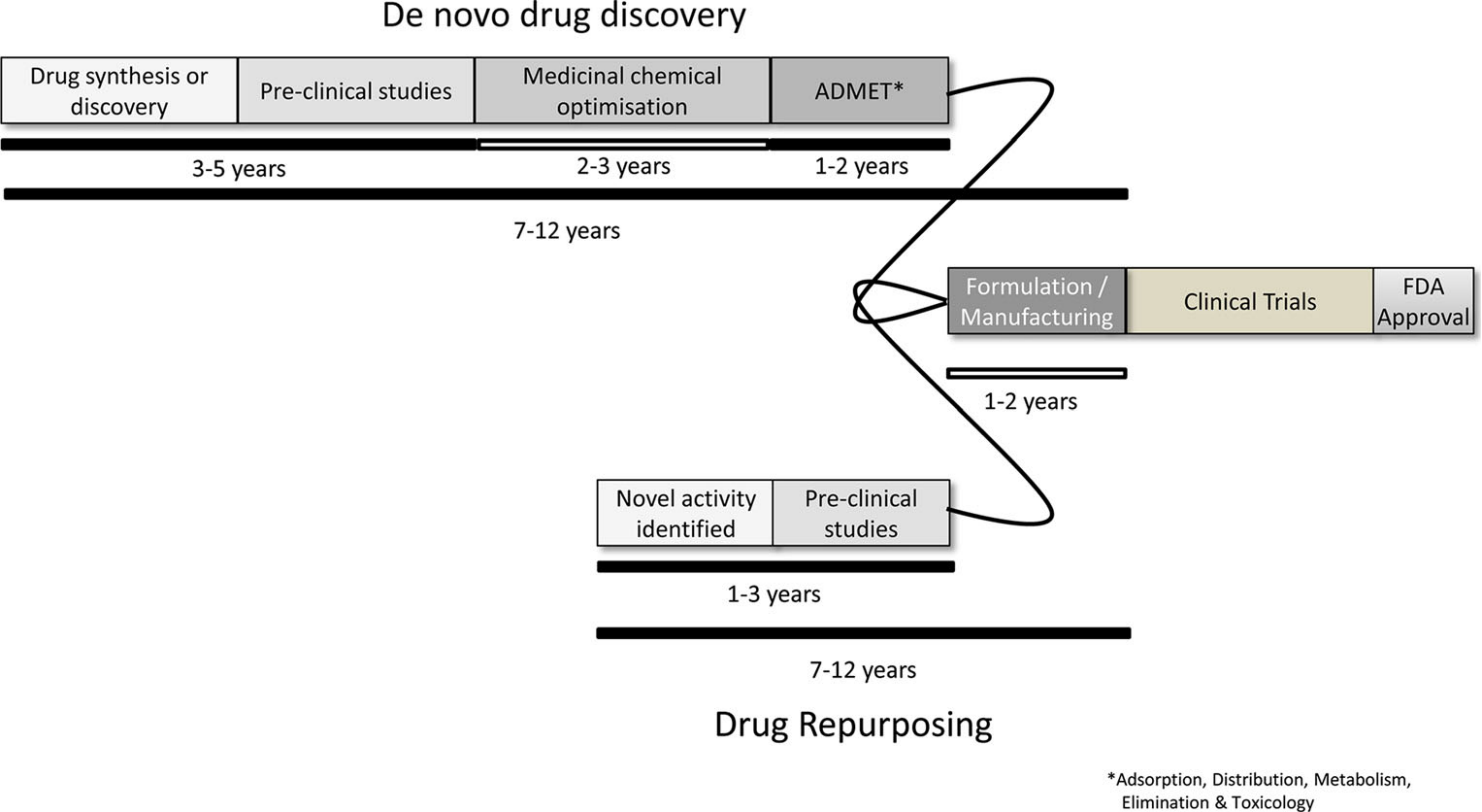
Comparison of the phases and timescale required for de novo drug discovery or repurposing drugs

In total, the time for drug discovery and development is around an average of 13 years of research and financial investment resulting in the drug being brought from the laboratory to a patient’s bedside (Fig. 1). The process of drug development entails multiple steps and phases of trials that attempt to seek FDA approval. The steps prior to clinical trials involve testing the efficacy, toxicity, and pharmacokinetic and pharmacodynamic profiles of the drug which usually take place by cell- and animal-based studies. Following the success of these steps, the drug will be then tested for efficacy and safety in human subjects, which usually occurs in three phases [6]:

- Phase 1 studies are focused on the safety of the drug.
- The drug will proceed to phase 2, only if it is not found to have unacceptable levels of toxicity, to test the effectiveness of the drug and its ability to successfully interact with its predetermined target. Usually in phase 2, patients with a specific disease are treated and often when refractory or resistant to previous therapies.
- Phase 3, the final stage, continues to evaluate safety and effectiveness but also tests the therapies against standard of care treatments or different doses often in a randomised trial basis. This phase usually requires significant numbers of patient subjects to allow for the generation of significant and reliable data to show significant benefit.

FDA approval is usually given following successful completion of phases 1, 2, and 3, which can take several years (Fig. 1). It is very common to see drugs fail to effectively target the disease in which they have been developed for or they are proved to be unsafe in humans and therefore do not receive FDA accreditation.

The pharmaceutical industry faces significant challenges, both politically and financially. In a study carried out by Tufts Center for the Study of Drug Development in 2014, the estimated cost to develop a new drug that gains marketing approval costs $2558 million, a significant increase compared to the previous study, in 2003, which estimated the cost to be $802 million [7]. This rise puts pressure on companies to insure drugs being developed do receive approval and results in the pricing of drugs rising significantly when in market, making them less affordable for health care centres and patients. Governments around the world are trying to contain costs, and, as health care budgets constitute a large part of governmental spending, these costs are the subject of intense scrutiny. In the USA, as elsewhere, drug costs are also the subject of intense political discussion [8].

### The demands of blood cancer

All cancers demand new treatments, and in this review, we focus on one specific type of cancer: blood cancers or haematological malignancies. There are four broad categories of blood cancers: leukaemia, myeloma, Hodgkin lymphoma and non-Hodgkin lymphoma. Together, these account for around 9 % of all cancers and are currently the fourth most common in both males and females in the world [9].

But these four categories contain over 60 different subtypes, as first highlighted in 2001, and then in 2008, when the World Health Organisation (WHO) produced an accepted classification of haematologic malignancy that defined each type according to immunophenotype, genetic abnormalities and clinical features [10, 11]. This was the first time that the genetics of the disease was combined with morphology, cytochemical and clinical information to define the classification of the disease. In the USA, it is estimated that a total of 157,000 people would have been diagnosed with a blood cancer in 2014, and that around 55,000 people would die in the USA of a haematology malignancy [12].

The high incidence and mortality rates highlight the need for the development of novel and effective treatments. It is also necessary that these drugs be introduced into the market at a much faster rate. Between 2005 and 2014, the FDA approved 268 new molecular entities (NMEs) [5], although only ~10 % of these NMEs had an indication for haematologic malignancies [13]. Overall, 23 of the 84 FDA cancer drug approvals in the past 5 years were associated with blood cancers (Table 1). Furthermore, of the cancer drugs which gained FDA approval in 2012, 11 were priced at more than $100,000 per patient per year, prices which are simply unaffordable to the majority of patients and almost all medical services [14].

**Table 1:** FDA haematology (cancer) approvals for 2011–2015

Drug	Year	Indicated blood cancer^a^	Previous approvals
Belinostat (BELEODAQ, Spectrum Pharmaceuticals, Inc.)	2014	Relapsed or refractory peripheral T-cell lymphoma (PTCL)	
Blinatumomab (BLINCYTO, Amgen Inc.)	2014	Philadelphia chromosome-negative relapsed or refractory B-cell precursor acute lymphoblastic leukaemia (R/R ALL)	
Bosutinib tablets (Bosulif, Pfizer, Inc.)	2012	Philadelphia chromosome positive (Ph+) chronic myelogenous leukaemia (CML)	
Brentuximab vedotin (Adcetris for injection, Seattle Genetics, Inc.)	2011	(1) Hodgkin lymphoma (2) systemic anaplastic large cell lymphoma (ALCL)	
Carfilzomib injection (Kyprolis, Onyx Pharmaceuticals),	2012	Multiple myeloma	
Erwinia chrysanthemi [Erwinaze, injection, EUSA Pharma (USA), Inc.]	2011	Acute lymphoblastic leukaemia (ALL)	
Ibrutinib (Imbruvica capsules, Pharmacyclics, Inc.)	2015	Waldenstrom’s macroglobulinemia (WM).	Previous approval (2014) for chronic lymphocytic leukaemia (CLL)
Ibrutinib (IMBRUVICA, Pharmacyclics, Inc.)	2014	Chronic lymphocytic leukaemia (CLL)	Previous approval (2014) for mantle cell lymphoma
Ibrutinib (IMBRUVICA, Pharmacyclics, Inc.)	2013	Mantle cell lymphoma (MCL)	
Idelalisib (Zydelig tablets, GileadSciences, Inc.)	2014	Relapsed chronic lymphocytic leukaemia (CLL)	
Lenalidomide capsules (REVLIMID, Celgene Corporation)	2013	Mantle cell lymphoma (MCL)	
Obinutuzumab (GAZYVA injection, for intravenous use, Genentech, Inc.; previously known as GA101)	2013	Chronic lymphocytic leukaemia (CLL)	
Ofatumumab (Arzerra Injection, for intravenous infusion; GlaxoSmithKline)	2014	Chronic lymphocytic leukaemia (CLL),	
Omacetaxine mepesuccinate (SYNRIBO for Injection, for subcutaneous use, Teva Pharmaceutical Industries Ltd.),	2012	Chronic myeloid leukaemia (CML)	
Oral suspension of mercaptopurine (Purixan, NOVA Laboratories Limited)	2014	Acute lymphoblastic leukaemia (ALL)	
Panobinostat (FARYDAK capsules, Novartis Pharmaceuticals)	2015	Multiple myeloma	
Pomalidomide (POMALYST capsules, Celgene Corporation)	2013	Multiple myeloma	
Ponatinib (Iclusig tablets, ARIAD Pharmaceuticals, Inc.)	2012	Chronic myeloid leukaemia (CML) or Philadelphia chromosome positive acute lymphoblastic leukaemia (Ph+ ALL)	
Rituximab (Rituxan, Genentech, Inc)	2011	Follicular, CD-20 positive, B-cell non-Hodgkin lymphoma	
Ruxolitinib (Jakafi, Incyte Corporation)	2014	Polycythemia vera (PV)	Previous approval (2011) for myelofibrosis
Ruxolitinib (Jakafi oral tablets, Incyte Corporation)	2011	Myelofibrosis, including primary myelofibrosis, post-polycythemia vera myelofibrosis and post-essential thrombocythemia myelofibrosis	
Vincristine sulfate liposome injection (Marqibo, Talon Therapeutics, Inc.)	2012	Philadelphia chromosome-negative (Ph−) acute lymphoblastic leukaemia (ALL)	

Until end of May 2015

^a^For further details on the Haematology/Oncology (Cancer) Approvals & Safety Notifications see:http://www.fda.gov/Drugs/InformationOnDrugs/ApprovedDrugs/ucm279174.htm

One of the earliest targeted therapies, imatinib, branded as Gleevec, saw its market price rise from £18,000 to £21,000 in the UK per patient per year. Gleevec is hugely successful and thus a profitable drug for the pharmaceutical company, Novartis, with an indication for the treatment of chronic myeloid leukaemia (CML) and many others. Whilst all the initial research and development cost had already been covered, the increased cost was probably based on its success.

Recognising the major issues associated with cancer in regard to the lack of effective treatments, multiple projects have been set up in an attempt to develop new processes to find novel therapies. One such project is the Repurposing Drugs in Oncology (ReDO) project [14].

The ReDO Project is a collaboration between Global Cures of the USA and Anticancer Fund in Belgium with the aim of the project is to find existing drugs which are non-cancer related and test their potential for becoming novel, repurposed treatments for cancers [14]. The success rate for new oncology drugs in phase I trials is 6.7 %, half the rate of non-oncological drugs [14]; therefore, the cost of oncology drug development impacts vastly on the health and global economy each year. So, the approach of drug repurposing should ease the pressure to contain costs by reducing the early-stage research and development costs associated with the current drug development model. One of the main concerns is the future economic impact the increasing incidence of cancer will have on the price of new therapeutic treatments. Costs for these treatments are increasing due to high failure rates and decreasing investment [15], whilst many approved drugs are not specific enough for the rare and mutated forms. Tumours become resistant to current treatments and displaying high levels of intra-tumour genetic heterogeneity, and so, treatments are probably acting as selective pressures [15]. For this reason, targeting treatments with single compounds will not be effective against resistant tumours. It is clear that new and repurposed drugs should be considered in combination with other targeting compounds against a tumour or be prescribed at earlier stages of the disease. Combinations between targeted therapies and traditional chemotherapeutic agents are now being widely used [16].

The ReDO project recognises that there are multiple sources of anticancer drugs apart from existing pharmaceutical armamentarium. The project intends to repurpose non-cancer drugs, which are well known, and find a new indication for these drugs as anticancer treatments [14]. In 2014, ReDO published an initial report addressing the current problems in oncology, stating the objectives of the project and the first six compounds which were considered to be potential candidates for repurposing. The six compounds were selected based on the fact that they are well known, generic drugs with well-detailed toxicology publications. The six compounds are mebendazole, nitroglycerin, cimetidine, clarithromycin, itraconazole, and diclofenac. To date, the project has published evidence for the anticancer activity of cimetidine, clarithromycin and mebendazole, with Mebendazole being evaluated for its effectiveness in leukaemia [17–19].

## Advantages of repurposing drugs

The most accepted argument in favour of this method of drug development is the reduced research and development time involved in bringing the drug to market and to the patient’s bedside. It has been estimated the development of novel drugs can take up to 17 years; whereas, the process of repurposing a drug may only be 3–12 years (Fig. 1) [1]. Repurposing drugs offers a high level of safety as there is already a wealth of accessible data describing the pharmacokinetics, toxicities, bioavailability, dosing, and protocols of the agents [16]. As ~30 % of trial drugs fail due to safety and repurposed, drugs hold an advantage of a reduced failure rate as they have already been tested for safety. Phase I clinical trials usually allow for dosage limits to be identified and may be avoided or use predetermined dosage schedules for the trial [16], and the cost of a relaunch is reduced compared to a launch of a novel compound.

### Potential barriers and limitations to drug repurposing

Some limitations have arisen when repurposing drugs. In various cases, a drug may show good efficacy for a new indication but at a much higher dosage than previously approved resulting in possible adverse effects relating to toxicity [16]. Research companies can be focused on certain disease areas, and so, potential indications for drugs can go untested [20]. Patent exclusivity can play a major role in limiting the potential of repurposing a drug. Drug patents are required by the developing pharmaceutical company so they are the only company able to manufacture, market, and avail of any profits made from the drug.

However, in the USA, a patent provides protection for up to 20 years, counting from the filing date. A similar length of 20-year patent protection is granted by the European Patent Convention (EPC). It is normal that a pharmaceutical company will apply for patency prior to commencing clinical trials. When a patent expires, the drug is termed ‘generic’ and so is available for other companies to manufacture and market. A good example of this process is paracetamol whose patent expired in 2007; since then, numerous versions have arisen and developed under many different brands [21].

The patent on the basic compound patent for Gleevec (imatinib) expired in the USA in July 2015, and in 2016, in major European countries, although for other polymorphic forms of Gleevec, the patent expires in 2019. There are also generic versions waiting to seek approval from the US Food and Drug Administration (FDA), which will be marketed at a much lower cost than the current price per patient per year. Litigation in 2014 resulted in Novartis permitting a subsidiary of Sun Pharma to market a generic version of Gleevec in the USA from February 2016 [22].

Current regulations relating to drug development can also act as a deterrent for repurposing drugs for some research groups. Strict legislations are involved when repurposing a patent drug. On-patent drugs can be screened for new indications without the need of chemistry and manufacturing applications or seeking the approval of the original owners providing that the product is used in agreement with the approved product label. In the case of off-patent or generic drugs, the new indication must be novel and have clinical benefits. For both of these indications, the new claim must not have been previously published or suggested in past literature. A major deterrent for research is the necessity to develop a new formulation so as to have the ability to achieve market exclusivity with the proposed new indication. An incentive for groups to take on this process is the Orphan Drug Act (ODA) which was set up in 1983 to encourage pharmaceutical companies to address the need for novel treatments for rare diseases [23, 24]. The European Union defines rare diseases as being those that affect less than 5 in 10,000 of the general population, whilst in the USA, an orphan disease affects less than 200,000 of the whole population. Haematological malignancies including AML, chronic myeloid leukaemia (CML) and multiple myeloma (MM) are known as rare and orphan diseases. The ODA offers pharmaceutical companies incentives to apply their research into developing treatments for such rare diseases.

Funding of grants and contracts may be given if the research is put towards an orphan disease. The FDA will also accept smaller patient numbers for trial when reviewing applications for orphan drug. Drug applications are usually associated with fees; however, in these causes, such fees are not claimed. If approval is awarded, a market exclusivity of 7 years in the USA and 10 years in the European Union is given under the ODA. Since the passing of the act in 1983 up until 2010, the FDA have approved 353 orphan drugs and granted orphan designations to 2116 compounds via the Office of Orphan Product Development (OOPD) of the FDA. This focus on orphan drugs and diseases has continued as of the 27 drugs approved by the FDA in 2013, 9 of these were for the treatment of rare diseases [20].

## Types of repurposing

In general, there are two simple concepts behind recognising the potential of a drug to be repurposed for a new indication. The first of these concepts is the identification of a drug to interact with multiple targets relatable to multiple diseases. These additional targets, i.e. off-targets, can arise serendipitously through screening or observed side effects. In some cases, these secondary targets cause the drug to be labelled as ‘dirty’ for the specified indication due to the undesired side effects produced. However, a certain target can prove beneficial for a new indication. Where a drug is found to have beneficial off targets, it can be known as being ‘desirably promiscuous’. The second concept is identifying multiple diseases, where one target is relevant to the progression of them all. The discovery of these new indications often occurs from a known target-based screen of a library of established and/or shelved compounds such as the Johns Hopkins library, which includes 3500 drugs.

To date, the most successful opportunity for drug repurposing arises serendipitously, most frequently due to observed side effects. Thalidomide is a prime example of such serendipitously observations leading to its repurposing for leprosy and multiple myeloma and explored further later in the review [25].

Drug repurposing can be applied at many phases of drug discovery and development but has a greater potential when the drug has already been tested for safety and efficacy.

Understanding the biology behind a disease is of particular importance when seeking to repurpose a drug. Knowing the mechanisms behind which a disease progresses is essential for identifying targets for its prevention. Imatinib was identified for new indications through studying its mechanisms (discussed later).

## Methods of drug repurposing

### Blinded search or screening methods

These types of methods are based on blinded searches without considering any known pharmacology or biological data and often result from serendipitous observations [26]. Blinded searches and screens have the advantage of flexibility associated with the application to a diverse number of target diseases [27]. Between 1999 and 2009, around 34 % of FDA-approved small molecules and biologics were identified via this method. Drugs repurposed beyond their labelled indications approved by the FDA using this method include sildenafil citrate for erectile dysfunction, rituximab in breast cancer, and etoposide for bladder cancer [28].

### Target-based methods

Target-based methods of drug repurposing involve high-throughput screening both, in vivo and in vitro, of drugs. The screening aims to identify a drug with a particular biomarker or protein of interest from a complex library of compounds [29]. The likelihood of a successful discovery of a potential drug is much higher than with the blinded screening method as most of the targets will link directly to the mechanisms of disease. Target-based repurposing allows for large libraries of drugs or compounds to be screened efficiently within a few days. This method is particularly popular with research groups when attempting to develop new treatments [30].

### Knowledge-based methods

A bio- or chemo-informatics analysis is applied in a knowledge-based method of drug repurposing. The utilisation of information already available from clinical trial information, drug-target networks, identified chemical structures of drugs and their target, and pathways involved in the drug activity is used in this method [31]. Researchers using the knowledge-based method avail of the published information to predict similarities in drugs and recognise potential new targets. This enables the identification of new targets for the drug, which would not be possible in the previous two methods. Drug repositioning has improved using this method as it is associated with a reduced failure rate especially if the drug has already been successful in gaining FDA approval [32].

### Signature-based methods

Gene signatures derived from disease ‘omics’ data with or without treatments are applied in signature-based methods to find unknown off-targets or unknown disease mechanisms [33, 34]. The volumes of genomics data available are increasing exponentially with advances in microarrays and next generation sequencing techniques. These advances are significant for the repurposing of drugs as genomic databases are continuingly built upon. Unknown mechanisms of drugs can be revealed via this method. Computational approaches are particularly important in signature-based methods [26]. Sirolimus was repurposed with this method for patients with acute lymphoblastic leukaemia with dexamethasone resistance by linking diseases treated by drugs by using gene signatures [35].

### Pathway- or network-based methods

Similarly to signature-based methods, pathway- or network-based methods of drug repurposing utilise disease omics data. In addition, this method uses available signalling or metabolic pathways and protein interaction networks to recreate disease-specific pathways that provide the key targets for repositioned drugs [36]. The main advantage of this method is the use of large amounts of diverse information to narrow general signalling networks to a specific network with few proteins/targets [32].

### Targeted mechanism-based methods

Targeted mechanism-based methods identify unknown mechanisms of drug action by combining information based on protein interaction networks, signalling pathways, and treatment omics data. This method is proving particularly important in studies where patients can develop resistance to a drug after initial success indicating that successful drug treatment must also include studies of the mechanism of drug action allowing better drug targets to be identified [30]. The significant advantage of this method is the potential to identify the mechanisms related to the treatment of drugs in their specific diseases [26]. Overall, the methods of drug repurposing share a common sequential process consisting of analysis, hypothesis generation and validation.

## Examples of repurposed drugs in blood cancers

### Thalidomide

Thalidomide was developed in the 1950s as a sedative and was used to alleviate morning sickness in pregnant women. However, it was noted that the drug could cause serious birth defects which led to the withdrawal of thalidomide from the market. During this time, it was estimated that at least 10,000 infants were born with malformations of the limbs and other body extremities in over 46 countries at this time [37]. In the later decades, researchers, such as pharmaceutical company Celgene, continued to test thalidomide for therapeutic effects. During this period of research by D’Amato, working in the lab of Judah Folkman, demonstrated that thalidomide significantly reduced neo-vascularisation [38]. Eventually, the drug was tested in patients with refractory multiple myeloma where it induced durable responses [39] through its ability to inhibit tumour necrosis factor alpha. The FDA approved thalidomide in 2006 as a treatment for multiple myeloma in combination with dexamethasone.

### Imatinib

It is common for cancer therapeutic drugs to be repurposed within the wider disease subtype they have been initially approved for. For example, imatinib mesylate, Gleevec, was originally approved for the treatment of chronic myelogenous leukaemia. But as imatinib is an inhibitor of the ABL kinase, it also showed to cross-reactivity with, and an ability to inhibit, KIT kinase. Mutations causing the activation of the KIT tyrosine kinase were identified as a cause in gastrointestinal stromal tumours (GISTs) [40], suggesting that imatinib be tested for efficacy against GISTs. Preclinical studies were conducted to tests the hypothesis of the potential indication and revealed imatinib did induce cell death in GIST cells which appeared to be related to inhibition of KIT kinase. These studies resulted in imatinib being approved of the treatment of GISTs [40]. Imatinib is now used for different blood cancer indications such as acute lymphoblastic leukaemia, chronic eosinophilic leukaemia and myelodysplastic/myeloproliferative neoplasms and also received approval for the treatment for systemic mastocytosis and dermatofibrosarcoma due to reaction with the platelet-derived growth factor receptor kinase [36].

### Dasatinib

Similarly to Imatinib, dasatinib was initially approved for the treatment of chronic myeloid leukaemia (CML) and repurposed for the treatment of patients with Philadelphia chromosome-positive acute lymphoblastic leukaemia (ALL) with resistance or intolerance to prior therapies. Dasatinib, manufactured under the name Sprycel as an oral tyrosine kinase inhibitor, is also in clinical trials for the treatment for glioblastoma [41] and has also been repurposed by network-based methods for breast cancer brain metastases by reconstructing the disease-specific networks to identify key targets [42].

## Improving the success of repurposing drugs

The success of drug repurposing is centred on its ability to improve efficacy. With the process availing of previously published toxicology and clinical information, there is a reduced risk of failure with the increased safety of the drug.

Along the complete process of novel drug development, there are multiple stages at which a drug can be predicted and validated to have an additional indication. As expected, the further along the progression of the discovery and developmental process, the more confidence can be associated with drug repurposing prediction. There are multiple different methods that can be applied along the timeline of drug development: the first of which is a large-scale approach involving gene profiling and molecular modelling. This first approach provides a low level of predictability due to the amount the candidates involved. Large pharmaceutical companies can apply this approach to every drug they develop. One limitation to large-scale approach is the need for complex technology usually inaccessible by smaller research and academic groups. Adding to the complexity of this approach is the decision on what to focus on in regard to repurposing the drug. A company can test individual drugs for second targets within the same indication or find a second indication for the drug. They can also seek to establish if the drug is effective in rare or orphan diseases (which can present with benefits to the company), an important decision is whether or not the company will consider all compounds available or only those that they have developed themselves. A second-step drug development relates to clinical trials and the observation of side effects experienced by treated patients. On observation of side effects of interest, separate clinical trials must be conducted to establish if the effect is reproducible.

An additional method, although much more time consuming than any previously discussed, is reviewing data of a patient who is prescribed with a particular drug of interest. This is a relatively simple approach to initial repurposing prediction through finding drug and disease relationships. Considering the scenario of a patient treated with a particular drug for one indication, it could be observed to have a therapeutic effect for any additional diseases the patient may possess and so present with a potential for repurposing investigations.

An important topic to cover when discussing drug repurposing is the need for animal models. The use of mouse models is believed necessary to the development of novel drugs. Mouse models are often used to test the efficacy of a compound. When the pharmacokinetics of a drug differs greatly between mouse and human trials, it can be impractical to mimic dosing schedules in humans. From this, it may be unjustified to approve the proceeding of human clinical trials for a new indication [13]. Some suggest animal models can be skipped if dosing remains constant in repurposing; however, the case is often not so simple and new indications can require different dosages which will require mouse models for efficacy testing.

## Future directions and conclusions

The understanding of disease pathology is continuingly growing through new technologies allowing us to specify the biological mechanisms behind the cause of the disease and its continued invasion. There are two popular approaches to improving the productivity of drug development: drug repurposing and personalised medicine. Drug efficacy plays a major role in drug failure. At least 30 % of drugs are failing due to poor efficacy [8]. Personalised medicine aims to treat patients based on grouping them with diseases into subtype-specific treatments in an attempt to improve drug efficacy. A major impact on efficacy is the complexity and heterogeneity of human diseases. Blood cancers, in common with other cancers, are groupings of many diseases each with subtypes some affected by gene expression profiles. Personalised medicine plans to diagnose patients and prescribes treatments which are specific and tailored to the molecular biology basis of the disease [43]. With increasing knowledge of the mutations and biology of diseases, personalised medicine is becoming increasingly evident and a future direction for patient treatment. The ability to sequence a person’s DNA means there is the potential to characterise a patient’s disease and its molecular composition and mechanism.

The goal of personalised medicine and the potential of drug repurposing are a powerful combination which share a common possibility of developing treatments for rare disease and those diseases termed ‘orphan’. Often the inadequate numbers of patients for rare diseases means that clinical trials are impossible due to the difficulty in recruiting sufficient numbers of patients [43]. Drug repurposing can deliver therapies to these patients, including those with blood cancers, in a more efficient and timely manner.

It is recognised that there are three key elements necessary for drug repurposing to have a successful future. The first of these is the availability of compounds and the information which is concerning safety and previous clinical studies. Furthermore, it is proposed that a list of compounds along with the associated information be made to all research and academic groups. The second element is to be open to investigating a broad indication space when screening for potential candidates. This can be carried out as one project group or multiple subgroups. Finally, the third key element is maximising and combining ideas through coupled or multiple collaborations between all sizes of research groups [20].

In order to aid continual repurposing research, it is essential that research groups have or have access to a physical collection of drug compounds. These collections of drugs or drug databases can be subjected to high-throughput screening. Some already established databases include The Rare Disease Repurposing Database (RDRD) and The National Clinical Guidance Centre (NCGC) Pharmaceutical Collection (NPC). The RDRD is a promising novel resource provided by the FDA [44]. This database publishes a comprehensive list of over 200 known products that have the potential to be repurposed to treat rare diseases. This resource is rich in information and offers sponsors a new tool for finding special opportunities to develop niche therapies for rare disease patients.

The NCGC-NPC is an established, broad, and open access database of approved and investigational drugs enabling repurposing and chemical genomics [20]. NPC Informatics Resource is available for virtual screening by any investigator with an internet connection. NPC is encouraging the sharing of drug collection, and in turn, the company requests researchers to share any findings and testing carried out with aid of the collection whether successful or not. Importantly, all the data collected from related studies will be made available publically [45].

In regard to haematologic malignancies alone, the Leukaemia and Lymphoma Society (LLS) has recently developed The Therapy Acceleration Program (TAP) with aim of speeding up the development of blood cancer treatments and supportive diagnostics [46]. The program also supports the drug repurposing projects. TAP comprises three divisions each with the sole aim of the program. The first division, the Academic Concierge, capitalises on the $63 million academic annual investment through grants. This division encourages further development of projects that have clinical promise. With disease and research experts in this division, projects are also aided through guidance in the drug development process and clinical trial and approval applications. The second division is The Biotechnology Accelerator. The main focus of this division is to identify and support small pharmaceutical companies that are developing novel blood cancer therapies but lack the expertise or technologic resources to allow for the full potential of the study and benefit patients. The final division, The Critical Trials Division, concerns the patients of blood cancers. This division provides patients with the tools to access relevant clinical studies. This highlights that for effective repurposing, a partnership is needed, a partnership that involves the research community, the pharmaceutical industry, regulatory authorities, charities and, perhaps above all, the needs of the patient.

## Conclusion

When it is considered that only 3 (7.3 %) of the 41 FDA-approved novel new drugs in 2014 [5] had indications for haematologic malignancies, it is obvious that new methods for drug development are necessary. Drug repurposing offers this opportunity with a decreased failure rate and lower costs. Some cases or repurposing leads to a new indication of a drug compound while others provide the opportunity to second-generation groups which are more potent and specific with the same indication. This method also offers the chance for small research and academic groups to participate in the drug development process.

It is evident that drugs have the potential to be repurposed at multiple steps. Drugs that have been withdrawn, approved, currently in clinical trials or have failed clinical trials all may have repurposing opportunities. Increasing the potential repurposing avenues is based upon the collaborations of diverse research groups and the sharing of new findings between charitable organisations, academic groups, and industries. It is expected that the assessment of a drug repurposing potential will become routine for every drug that has passed through a phase I clinical trial and already approved drugs. Often, blood cancer drugs are repurposed for other cancer indications or additional haematological malignancies; very few are repurposed as novel treatment of blood cancers from other disease. However, as a note of caution, any attempt to identify new therapeutic treatments for hematologic malignancies from repurposed drugs will still require detailed analysis of the mechanisms behind the drugs activity. But repurposing will contribute to improving and increasing the therapeutic armoury available for blood, and other, cancers.
